# Adenoid cystic carcinoma of distal trachea: A case report

**DOI:** 10.1002/ccr3.8495

**Published:** 2024-02-17

**Authors:** Sang Gyu Choi

**Affiliations:** ^1^ Department of Radiation Oncology Dankook University Hospital, Dankook University School of Medicine Cheonan Chungcheognamdo Korea

**Keywords:** adenoid cystic carcinoma, case report, CCRT, distal trachea

## Abstract

**Key Clinical Message:**

This case report highlights the rarity of distal tracheal adenoid cystic carcinoma and emphasizes the importance of considering it as a differential diagnosis in patients presenting with sustained dyspnea. Early diagnosis and a multidisciplinary approach involving thoracic surgeons, radiation oncologists, and medical oncologists are crucial for optimal treatment planning and patient outcomes. Further research is warranted to better understand the pathogenesis, molecular characteristics, and optimal management strategies for this rare tracheal malignancy.

**Abstract:**

Adenoid cystic carcinoma (ACC) is an unusual malignant neoplasm that mostly arises in the minor salivary glands. It can also occur in various mucous membrane‐lined structures, including the trachea. The treatment of choice is surgery in resectable cases, but radiotherapy and chemotherapy are generally utilized for unresectable cases or palliative treatment. In this study, I report my experience with a case of unresectable ACC occurring in a distal trachea treated with concurrent chemoradiotherapy (CCRT), based on the patient's clinical presentation, diagnostic workup, histopathological findings, treatment modalities, and long‐term prognosis.

## Graphical abstract

Pre‐treatment finding revealed that a large polypoid intra‐luminal mass, arising proximal to carina protruding in to the lumen and near complete obstruction of lumen

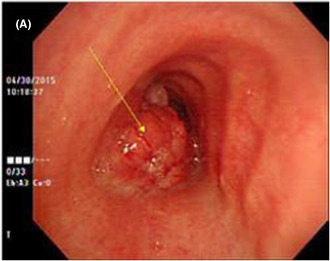

## 1 INTRODUCTION

The incidence of tracheal tumors is very rare, with less than 0.2 cases per 100,000 people per year, and ACC accounts for around 10% of them.^1^, ^2^ The most common are squamous cell carcinoma (SCC) and adenoid cystic carcinoma (ACC). Adenoid cystic carcinoma (ACC) is an uncommon malignant neoplasm that accounts for less than 1% of all malignant tumors, with approximately 30% of cases arising in the head and neck region, especially the salivary glands.^3^ Typically arising in the salivary glands, this cancer type has been documented in various anatomical sites, albeit infrequently. To date, there is limited documented literature on ACC of the trachea,^3^ making each case of particular interest for its clinical and therapeutic implications. Surgery is recommended as the treatment of choice for tracheal ACC, and historical case reports have focused on the surgery or surgery followed by radiotherapy that controls microscopic disease such as close or positive resection margin. In very rare cases for unresectable patients, the exact role of radiotherapy (RT) or CCRT remains controversy.^2^, ^4^

This case report focuses on a compelling and complex case of ACC located in the distal trachea extending to the left main bronchus, carina, and abutted to the esophagus. I aimed to provide a comprehensive account of the patient's clinical presentation, the intricacies of diagnostic evaluation, the employed treatment modalities, histopathological findings, and the patient's long‐term prognosis. Furthermore, it emphasizes the significance of a multidisciplinary approach to address this rare and potentially aggressive malignancy and offers insights into enhancing the management of ACC in atypical anatomical locations.

## 2 CASE PRESENTATION

A 52‐year‐old man presented to our hospital with a two‐month history of progressively worsening dyspnea and blood‐tinged sputum in April 2015. He occasionally complained of coughing, but there was no weight loss. Since his early 20s until he was diagnosed with cancer, his medical history was unremarkable other than smoking half a pack of cigarettes a day and drinking a bottle of alcohol a week.

Physical examination revealed decreased breath sounds over the left lower chest. On laboratory examinations, no abnormality was found in routine blood and urine analyses.

### 2.1 Diagnostic assessment

**FIGURE 1:**
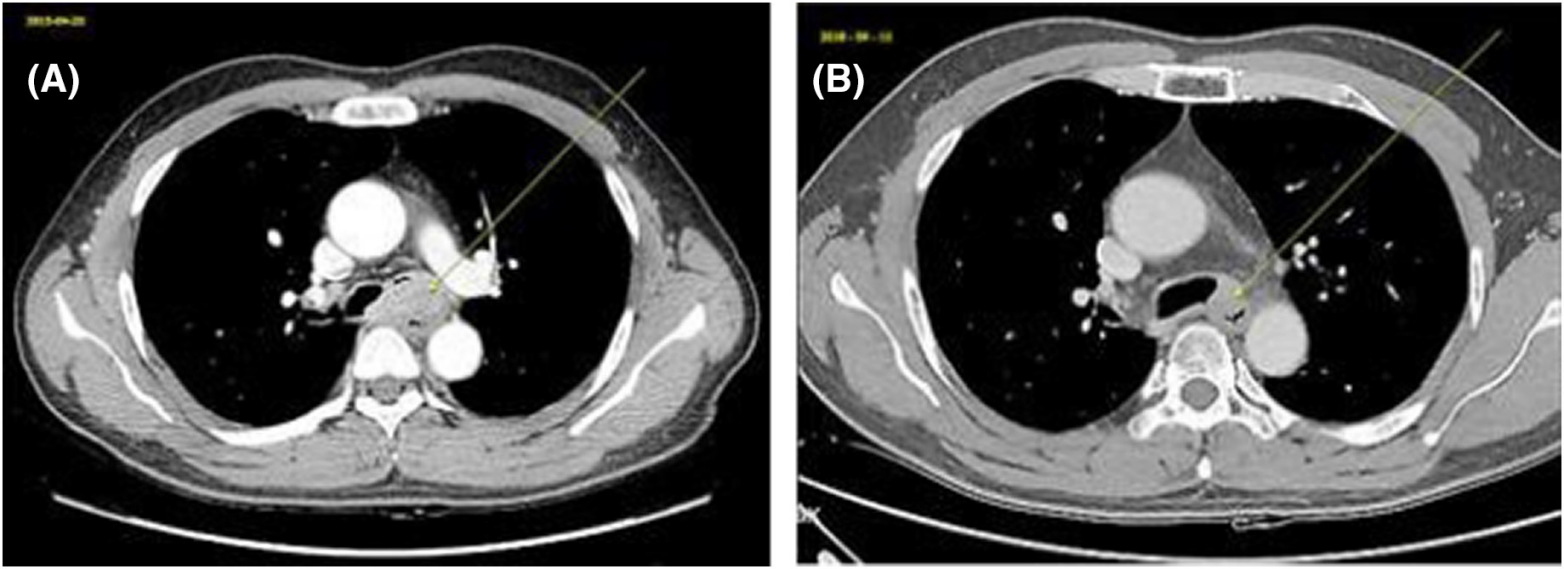
Chest computed tomography (CT) scan. (A) Pretreatment Chest CT scan showing tumor located at the carina level in the distal trachea with total obstruction to the left main bronchus and partially pushed right main bronchial lumen (arrow)—axial view. (B) After 3 years of completion of CCRT, Chest CT scan showed initial large polypoid mass was significantly decreased in size.

**FIGURE 2:**
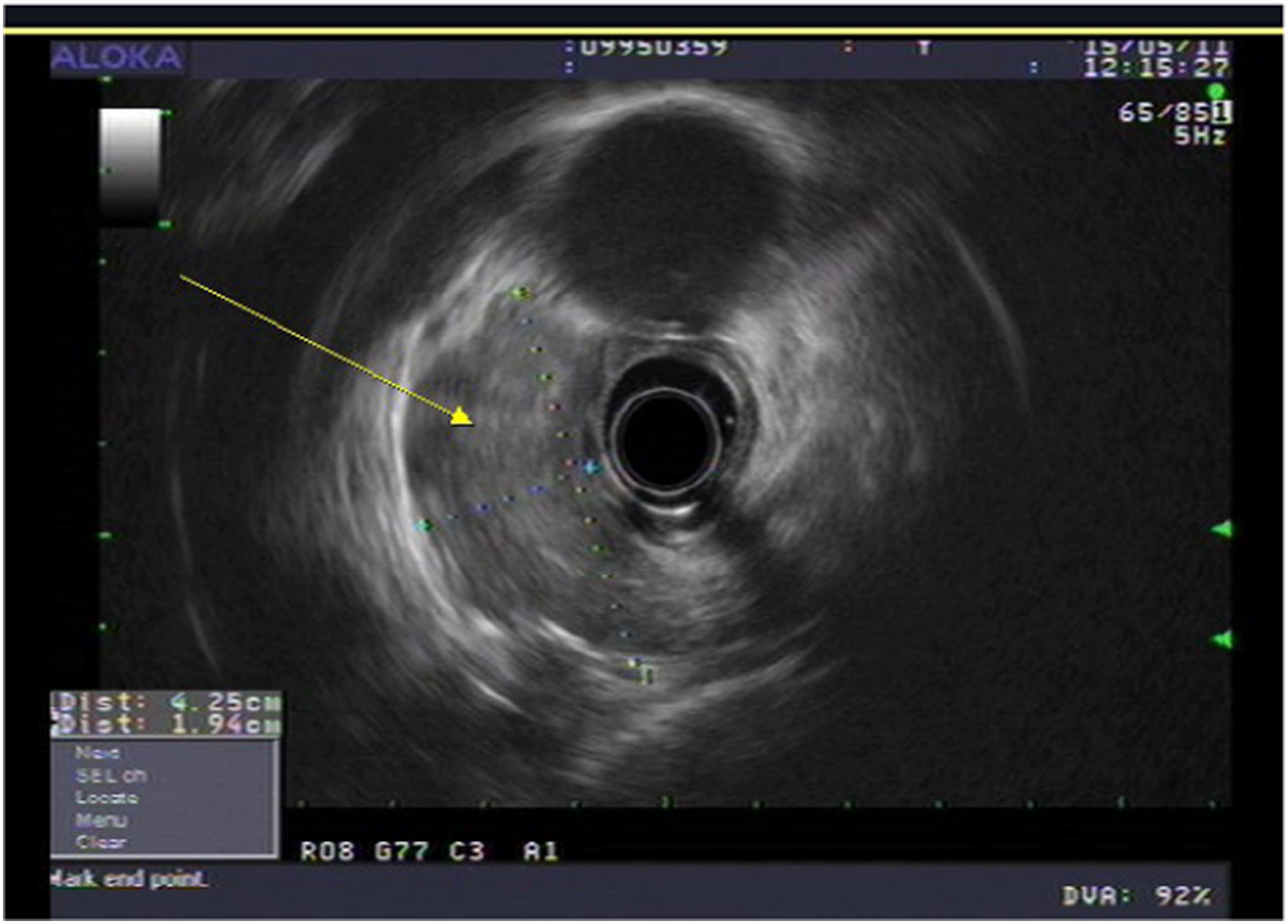
Endoscopic ultrasound (EUS) endoscopic ultrasound presented a 43 × 23 mm‐sized cancer lesion ranging from UI 33 cm to 29 cm. On EUS, the adventitia layer could not be distinguished, and esophageal invasion was strongly suspected as focal discontinuity of the SM layer and thickening of the PM layer were observed. (arrow).

**FIGURE 3:**
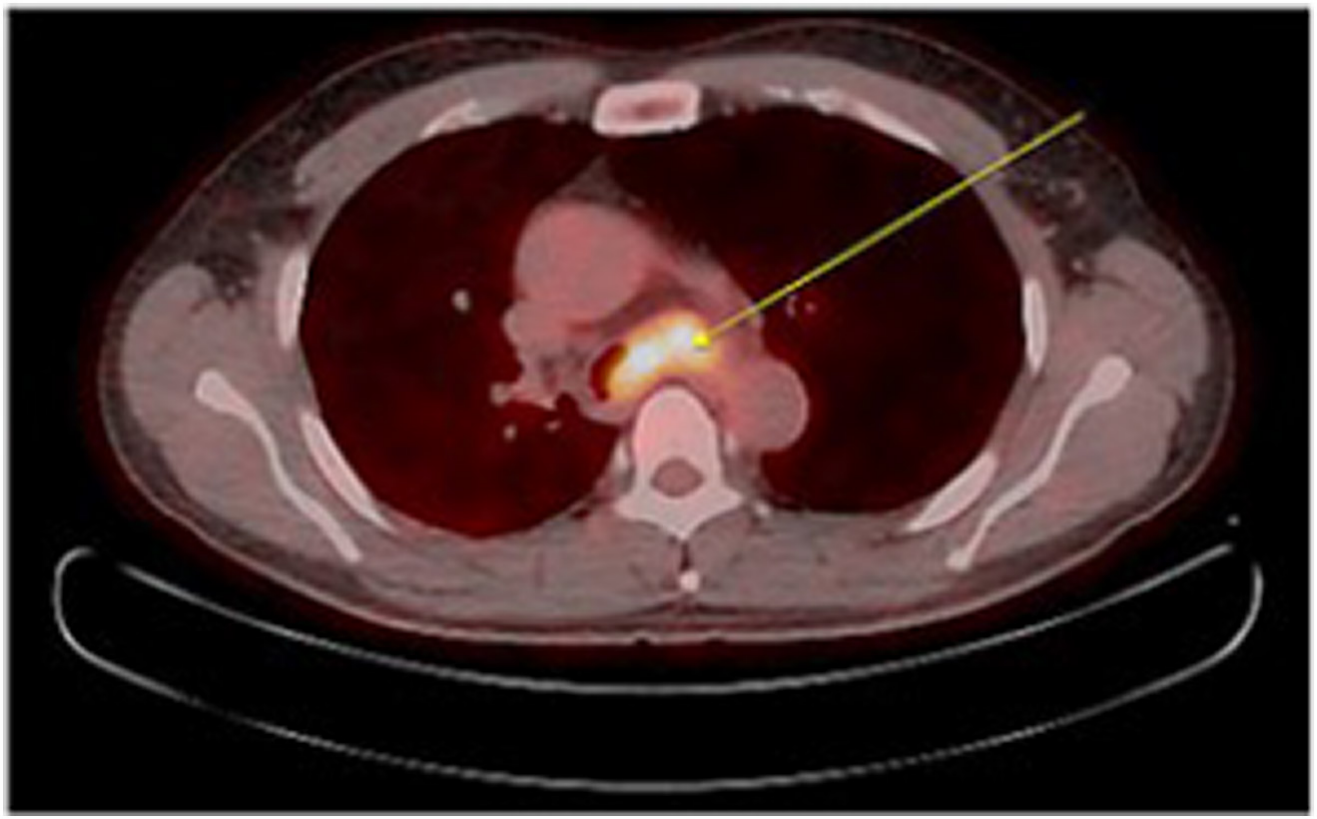
Pretreatment positron emission tomography / computed scanning demonstrating a hypermetabolic lesion (SUVmax. 4.3) in the distal to carina level left main bronchus (arrow)—axial view.

**FIGURE 4:**
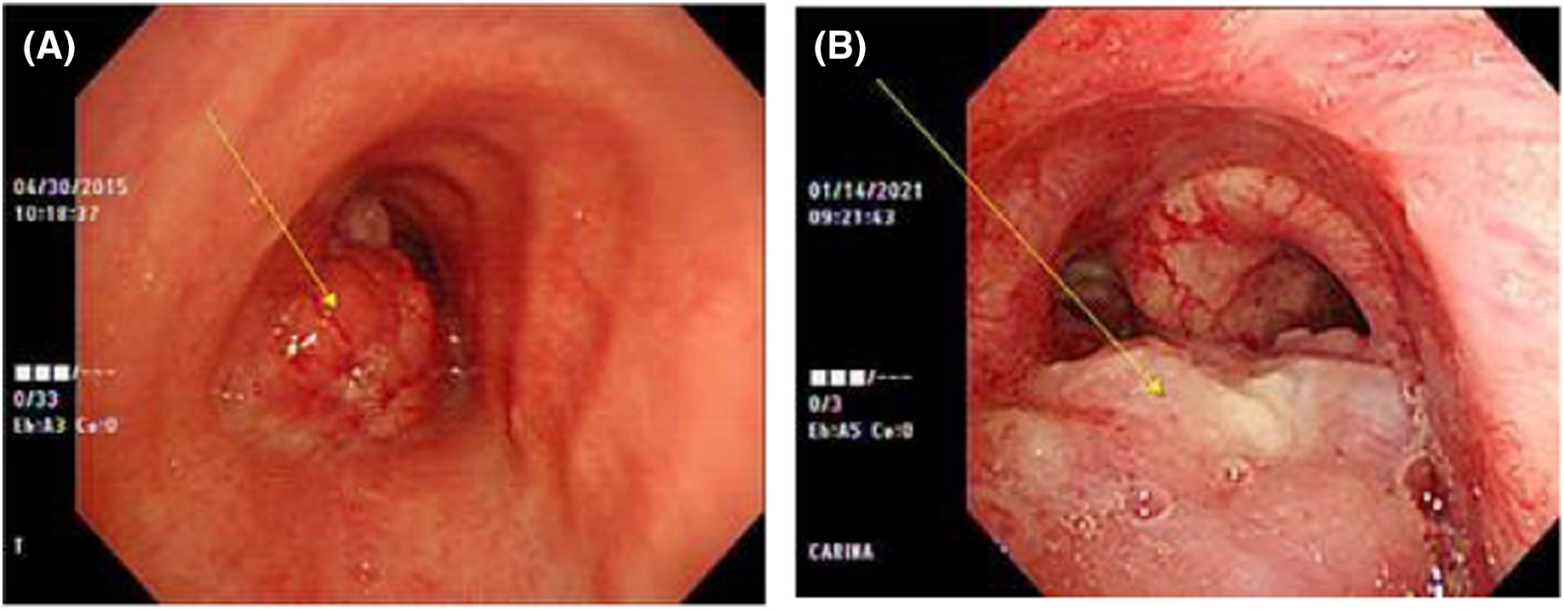
Bronchoscopic findings. (A) Pretreatment finding revealed a large polypoid intra‐luminal mass, arising proximal to the carina protruding into the lumen and near complete obstruction of the lumen; (B) After 6 years of completion of CCRT, bronchoscopy indicated that the previous large polypoid intra‐luminal mass was significantly eliminated.

**FIGURE 5:**
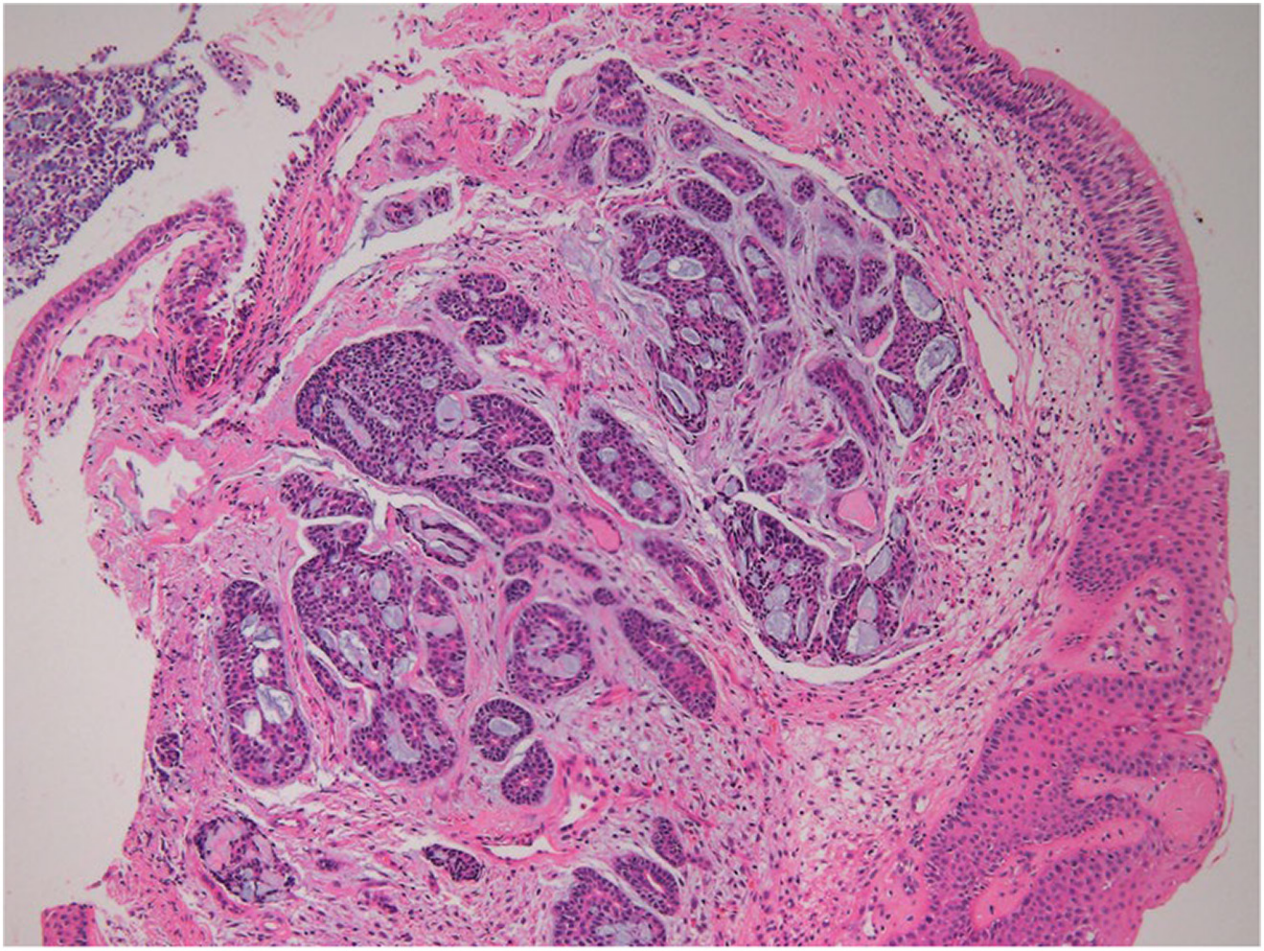
Histopathologic finding showing heterogeneous growth pattern with cribriform type is predominant adenoid cystic carcinoma. H and E, ×100.

Initial chest X‐ray showed a huge mass obstructing mass in the carinal area, followed by chest computed tomography (CT) demonstrated about 4.3 cm‐sized irregular heterogeneous enhancing mass in the distal trachea to proximal left main bronchus with involvement of carina and proximal right main bronchus and near complete obstruction of left main bronchus with invasion into surrounding mediastinum. Mass is widely abutting with esophagus and invasion cannot be excluded (Figure 1A). To rule out esophageal invasion, an endoscopy was done, but there was no direct esophageal mucosal invasion and followed endoscopic ultrasound (EUS) showed 43 × 23 mm‐sized mass located at the mid esophageal level, from the upper incisor (UI) 33 cm to 29 cm, and impossible to distinguish the adventitia layer with focal invasion of the muscle layer, the esophageal invasion was strongly suspected (Figure 2). Further staging with positron emission tomography (PET) revealed the absence of distant metastasis (Figure 3). The tumor was staged as T4bN0M0 according to the American Joint Committee on Cancer (AJCC) staging system. The patient was submitted to a bronchoscopy, revealing an irregular, polypoid lesion with involvement of the tracheal submucosa (Figure 4). A biopsy was performed using bronchoscopy, and histopathological analysis revealed a diagnosis of adenoid cystic carcinoma (Figure 5). Immunohistochemical analysis showed that c‐KIT(CD117), E‐Cadherin, CK5, and p63 are positive, and Ki‐67 is a focally positive result. The patient was diagnosed with adenoid cystic carcinoma of the distal trachea with staging cT4bN0M0.

### 2.2 Treatment

**FIGURE 6:**
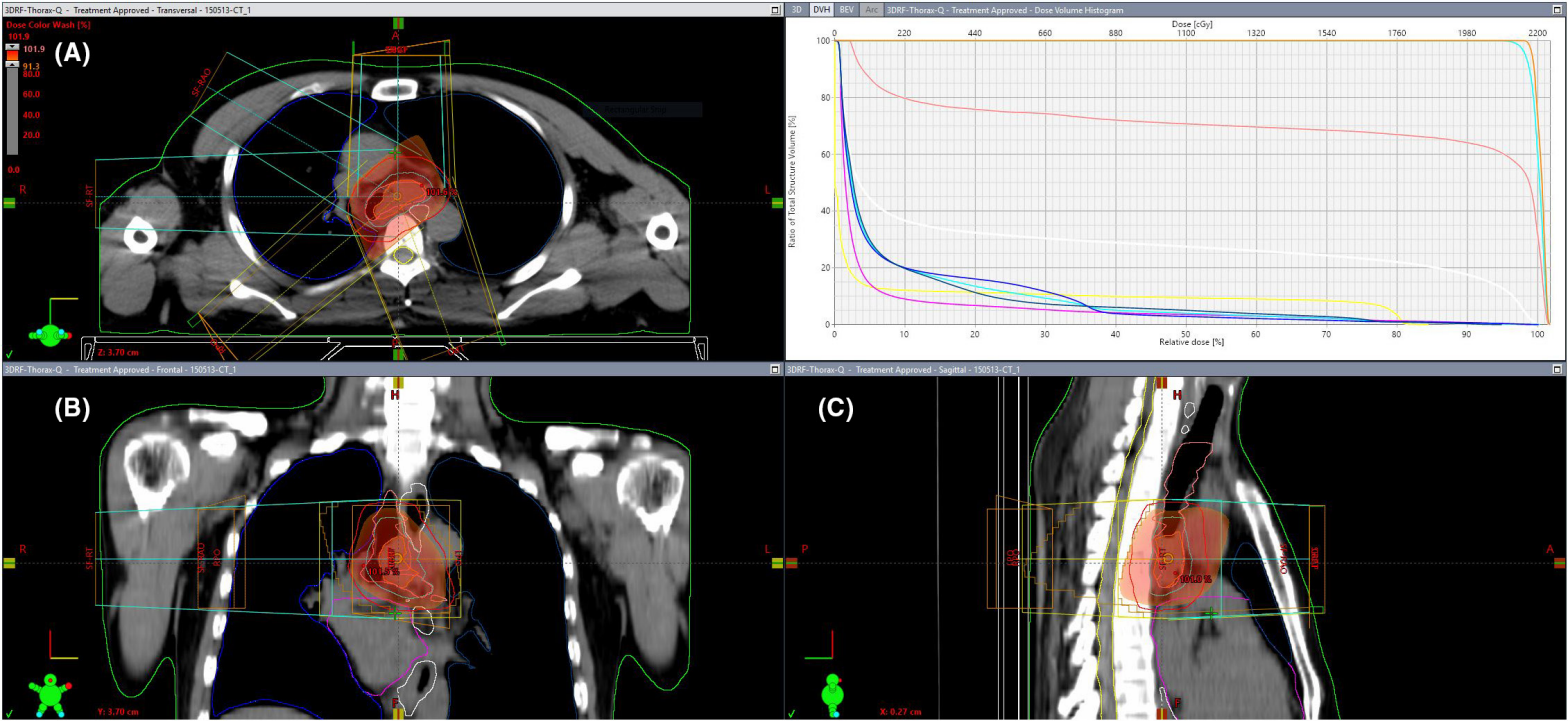
Radiotherapy plan presented in computed tomography scans of the treated regions with 3D reconstruction. Isodose levels are shown in the dose color wash method around 6600 cGy (red line) and DVH (dose volume histogram). (A) Axial view; (B) Sagittal view; (C) coronal view.

Multidisciplinary consultation involving thoracic surgeons, radiation oncologists, and medical oncologists was obtained to discuss treatment options. Given the extent of the tumor with esophageal invasion, concurrent chemoradiotherapy (CCRT) was considered the primary treatment modality. Conventional 3‐dimensional Radiotherapy (3DRT) was delivered with 6 megavoltage (MV) X‐rays. The target volume consisted of the primary tumor with proper margin, the planned dose was a total of 6600 cGy/33 fx to the planning target volume, and a fraction size of 2.0 Gy was given five times each week for 6.5 weeks from May 18 to July 6, 2015 (Figure 6). Concurrent chemotherapy was combined cisplatin with docetaxel, #2 cycles during radiotherapy, followed by # 14 cycles was delivered. Follow‐up study was performed every 6 months after completion of CCRT, including chest CT, 1 year after completion of CCRT, the response was PR (partial response) that significant decrease in mass size to pre‐CCRT initial chest CT, but further change over the next 5 years. January 2021, a follow‐up study includes chest CT revealed local progression, confirmed by bronchoscopic biopsy (Figures 1B and 4B). After the discovery of progression, the patient went to another hospital and no further follow‐up was performed.

## 3 DISCUSSION

Tracheal adenoid cystic carcinoma (ACC) is an extremely rare malignant tumor that arises from the salivary glands. While ACC usually occurs in the head and neck region, its occurrence in the trachea is exceedingly uncommon. Tracheal tumors can arise from the primary site itself or results of direct invasion from nearby structures. Tumors of the trachea grow slowly and asymptomatically, often without any unusual symptomatic presentation until they occupy 75% of the cross‐sectional area of the airway.^5^, ^6^ Primary trachea tumors are classified as benign and malignant and malignant tumors are more common in adults and consist of squamous cell carcinoma, followed by adenoid cystic carcinoma.^7^

The location of tumor origin in the trachea varies between reports, but to summarize, cancers located in the distal trachea are predominantly squamous cell carcinoma (SQCC), whereas ACC originates in the proximal trachea. Kim et al. reported that only 2 out of 13 patients had cancer located in the distal trachea mainly the left main bronchus; Je et al. reported that 7 out of 22 patients had cancer located at the carina level involved the main bronchus. In a study by Jiang et al., the distribution of ACC according to location was upper 6, middle 1, and lower 3, with most cases being located at the upper part, in contrast, 14 out of 19 SQCC patients showed located at the distal part. In comparison with the malignant tumors located in the upper one‐third, those in the lower trachea showed worse survival outcomes.^2^, ^8^, ^9^ In my case, the tumor was located in the far distal left main bronchus, invaded the carina, and was strongly suspected to have invaded the outer esophageal wall on various imaging studies. These findings show that this patient is a very exceptional case that deviates from the usual features.

Squamous cell carcinoma is male‐dominant and occurs in older age group like 60–70 years old. It commonly has a history of smoking, rapid growth, and biologically aggressive, so the usual diagnosis is obtained within 4–6 months of symptom onset. ACC of the trachea accounts for less than 10%–15% of all tracheal malignant tumors.^10^ Its biological behavior is resembling the salivary tumor in the head and neck regions. Although it has a low degree of malignancy and a relatively slow growth rate, it has a high recurrence rate and is characterized by direct invasion into surrounding tissues, and generally infiltrates through the submucosal layer or around nerves.^11^ ACC is usually not associated with smoking, has sexually equal prevalence and younger than SQCC, and slow growing, so diagnostic confirmation is later than SQCC.

The clinical presentation of tracheal ACC can vary, but the most common symptom is dyspnea, which is often sustained and progressively worsening, as seen in our case report. Other symptoms may include cough, hemoptysis, wheezing, stridor, or recurrent respiratory infections, depending on the location and extent of the tumor.^12^, ^13^, ^14^ Because of these non‐specific respiratory symptoms often misdiagnosed as asthma or bronchitis, leading to a delayed diagnosis.

Accurate diagnosis of tracheal ACC is essential for appropriate management. Diagnostic evaluation typically involves imaging studies, such as computed tomography (CT) and magnetic resonance imaging (MRI), to assess the size, extent, and location of the tumor. Additionally, bronchoscopy with biopsy is crucial for obtaining tissue samples for histopathological analysis.^3^

Histopathological examination of the biopsy specimens is necessary to confirm the diagnosis of ACC. Microscopically, ACC displays characteristic histological features of cribriform, tubular, and solid type. The most common is the cribriform type, characterized by island‐like clusters of tumor cells in the tubular structure that are sometimes filled with mucin. Tubular types are the most differentiated and have the best prognosis. Solid types indicate poor prognosis and risk of distant metastasis including lung. Immunohistochemical staining can aid in confirming the diagnosis, with positive staining for markers of ductal and myoepithelial/basal cells such as smooth muscle actin(SMA), p63, Ki‐67, and S100 are expressed by ACC and CD117.^15^, ^16^ In my case, it showed typical cribriform types, and immunohistochemical staining results are c‐KIT(CD117), E‐Cadherin, CK5, and p63 are positive, and Ki‐67 is a focally positive result.

Staging of tracheal ACC is important for treatment planning and prognosis. However, due to the rarity of tracheal ACC, there is no specific staging system tailored for this particular tumor. Therefore, the AJCC staging system for salivary gland ACC is often applied. Recently, one study showed the prognostic difference in cases who were classified use by TNM classification, which suggests that TNM classification may also be useful in tracheal cancer.^17^ According to TNM classification, in my patient's case, the cancer invaded the carina and Lt. main bronchus and abutted the esophagus by EUS, so esophageal invasion was strongly suspected, so it was categorized as T4bN0M0 by Macchiarini proposal.^18^

The primary treatment modality for localized tracheal ACC is surgical resection.^12^ The goal of surgery is to achieve complete resection with negative surgical margins. The extent of resection depends on the size and location of the tumor and may involve segmental tracheal resection with primary anastomosis or sleeve resection. In cases where complete resection is not feasible, palliative procedures such as debulking surgery or tracheal stenting may be considered to alleviate airway obstruction and improve symptoms.^3^, ^14^

Radiotherapy is routinely indicated for cases with ACC due to its propensity for local recurrence in head and neck cancer. The indications for postoperative radiotherapy include positive/close margin, incomplete resection, the presence of perineural invasion, and positive L/N involvement.^19^, ^20^, ^21^, ^22^ Postoperative radiation therapy has been associated with improved local control and disease‐free survival rates in some retrospective studies,^23^ but some trials have shown no survival benefit despite a benefit in locoregional control.^24^, ^25^, ^26^ In unresectable or medically inoperable cases, radiotherapy alone or chemoradiotherapy is recommended as in my case, but its effectiveness is not clear.^2^, ^27^

The optimal dose and fractionation schedule for radiation therapy in tracheal ACC are not well‐established, and treatment decisions should be individualized based on factors such as the extent of resection, the presence of positive margins, and patient‐specific considerations. Postoperatively, about 60 Gy to the R0 resected primary tumor bed, 66 Gy in the setting for close or positive margin and unresectable cases, about around 70 Gy setting, it is reasonable to extrapolate from other head and neck ACC.^28^, ^29^

The role of chemotherapy or CCRT in the management of tracheal ACC is very limited due to its rarity, but it is reasonable to extrapolate from salivary gland malignant tumors. High‐risk salivary gland cancer underscores the need for systemic therapy, such as chemotherapy that addresses the high rate of distant metastasis must be included in the improvement of clinical results. Its rationale also draws from squamous cell carcinoma in head and neck treatment. There are very few studies of CCRT used cisplatin‐based combination regimens for high‐risk salivary cancer,^30^, ^31^, ^32^ making the evaluation of gain of CCRT in this group controversial, and no randomized prospective studies have been performed, making this treatment option still controversial. Fortunately, an ongoing randomized phase II/III study (RT0G 1008) of adjuvant CCRT versus RT lone in resected high‐risk malignant salivary gland tumors currently under investigation includes high‐grade ACC (solid component >30%). The difference in prognosis between treatment modalities is that radiation alone is not as curative as surgery, and while some studies have reported no difference between surgery alone and surgery plus radiation, most physicians prefer to add radiation after surgery.^6^ In the case of tracheal ACC, there are not many reports due to the small number of patients, but the study by Gelder et al, which reported on a relatively large number of patients, and some case reports show relatively good results with a 5‐year survival rate of 33%–100% and a 10‐year survival rate of 50%–58%, and a report by Gaissert et al., comparing survival rates with resectability, showed mean survival time was 69 months with resected ACC, and 41 months with unresectable ACC. Survival rates were 52.4% and 29% 5‐ and 10‐year survival, respectively, in resected ACC, but only 33.3% and 10% 5‐ and 10‐year survival in unresectable ACC of the trachea.^6^, ^33^, ^34^ In my case, after being first diagnosed with ACC of distal trachea in 2015, CCRT was performed and PR was achieved without serious side effects for 6 years until local progression was observed in 2021. Recently, there has been growing interest in immune checkpoint inhibitors known as monoclonal antibody (MAB) can be designed to target for checkpoint (act like switches that need to be turned on or off to start an immune response). Immune checkpoint inhibitors do not kill cancer cells directly but work by helping the immune system to better find and attack the cancer cells. The immune response is regulated by the number of checkpoints with PD‐1 (programmed cell death proteins 1), PD‐L1 (programmed death ligand 1), and cytotoxic T‐lymphocyte‐associated antigen 4 (CTLA‐4). Some checkpoint inhibitors (PD‐1 inhibitor; Keytruda, PD‐L1 inhibitor; Tecentric) have been shown to be helpful in treating many different types of cancer. In ACC, resistant to immune checkpoint inhibitors due to low tumor immunogenicity and lack of PD‐L 1 expression. Instead, various clinical studies have focused on PSMA (Prostate‐specific membrane antigen), which is physiologically expressed in a variety of tissues, including the prostate, lacrimal gland, salivary gland, kidney, small intestine, and central and peripheral nervous system. The high expression of PSMA in ACC offers several possibilities as a targeted therapy and warrants further clinical investigation.^35^

## 4 CONCLUSION

This case report highlights the rarity of distal tracheal adenoid cystic carcinoma and emphasizes the importance of considering it as a differential diagnosis in patients presenting with sustained dyspnea. Early diagnosis and a multidisciplinary approach involving thoracic surgeons, radiation oncologists, and medical oncologists are crucial for optimal treatment planning and patient outcomes. Further research is warranted to better understand the pathogenesis, molecular characteristics, and optimal management strategies for this rare tracheal malignancy.

## AUTHOR CONTRIBUTIONS

**Sang Gyu Choi:** Conceptualization; data curation; formal analysis; funding acquisition; investigation; methodology; project administration; resources; software; supervision; validation; visualization; writing – original draft; writing – review and editing.

## FUNDING INFORMATION

This article did not receive any grants.

## CONFLICT OF INTEREST STATEMENT

The authors declare no conflicts of interest.

## CONSENT

Written informed consent was obtained from the patient to publish this report in accordance with the journal's patient consent policy.

## Data Availability

All the findings are present within the manuscript.
